# A Randomized Controlled Trial of Unresisted vs. Heavy Resisted Sprint Training Programs: Effects on Strength, Jump, Unresisted and Resisted Sprint Performance in Youth Rugby Union Players

**DOI:** 10.5114/jhk/200121

**Published:** 2025-01-31

**Authors:** Santiago Zabaloy, Robin Healy, Lucas A. Pereira, Eduardo Tondelli, Luciano Tomaghelli, Juan Aparicio, Franco Vega, Joaquín Medrano, Julián Giráldez, Thomas Comyns, Tomás T. Freitas, Irineu Loturco

**Affiliations:** 1 Faculty of Physical Activity and Sports, Universidad de Flores, Buenos Aires, Argentina.; 2 Department of Human Movement Sciences, Federal University of São Paulo, São Paulo, Brazil.; 3 Rugby Department, Belgrano Athletic Club, Buenos Aires, Argentina.; 4 NAR—Nucleus of High Performance in Sport, São Paulo, Brazil.; 5 Department of Health Sciences, Technological University of the Shannon, Athlone, United Kingdom.; 6 Faculty of Medicine, University of Buenos Aires (UBA), Buenos Aires, Argentina.; 7 Faculty of Medicine, Universidad del Salvador, Buenos Aires, Argentina.; 8 Department of Physical Education and Sport Sciences, University of Limerick, Limerick, Ireland.; 9 UCAM Research Center for High Performance Sport, UCAM Universidad Católica de Murcia, Murcia, Spain.; 10 Facultad de Deporte, UCAM Universidad Católica de Murcia, Murcia, Spain.

**Keywords:** athletic performance, team sports, youth athletes, sprint speed, resistance training

## Abstract

This study aimed to compare: 1) the effects of a 4-week unresisted vs. resisted sprint training programs (UST and RST with 50% body mass, respectively) on both resisted and unresisted sprint performance; and 2) the effects of these sprint training schemes on various strength-power measures (i.e., one-repetition maximum [1RM] and the isometric squat test (ISqT), eccentric hamstring strength in the Nordic hamstring exercise [NHE], and vertical and horizontal jump distances). Thirty-five under-19 male academy rugby players participated in the study and were randomly assigned to one of the two training groups. Players’ unresisted and resisted (50% BM) 30-m sprint performance, squat 1RM, ISqT, NHE, and jump capabilities were tested on different occasions. Only UST produced a significant reduction in unresisted 30-m sprint time (p < 0.05), whereas both groups exhibited significant changes in resisted sprint times at 10 m and 30 m, as well as maximum velocity (p < 0.005; ES: large). Regarding strength measures, RST led to significant increases in ISqT peak force, horizontal jump distance, and NHE strength (p < 0.011; ES: large). Overall, no significant differences were detected between UST and RST in any of the primary or secondary measures after the intervention. Both training methods were equally effective in improving resisted sprint performance in youth male rugby players. Moreover, UST and RST could be effective options for maintaining or even improving various neuromuscular measures (e.g., dynamic-explosive, isometric, and eccentric strength) when lower limb resistance training is reduced during the competitive season due to the congested schedule.

## Introduction

Improving sprint performance is a major objective of most physical preparation programs across a variety of sports, such as track-and-field and field-based team-sports (e.g., rugby and soccer) (Nicholson et al., 2021; Stavridis et al., 2023; Zabaloy et al., 2023). Specifically in rugby, not only sprinting speed but also sprint momentum plays a crucial role in gaining an advantage during contact situations (Jones et al., 2018; Zabaloy et al., 2021). For this purpose, resisted sprint training (RST) has been identified as an effective method because it provides mechanical overload (by reducing sprinting velocity), which requires the generation of high horizontal ground reaction forces and increases impulse against the supporting ground (Alcaraz et al., 2018; Petrakos et al., 2016; Stavridis et al., 2023). RST employs a wide range of tools (e.g., sled pushing and pulling, parachutes, weighted vest, sand dunes, etc.) that can be used to enhance sprint performance. On the other hand, unresisted sprint training (UST) is a highly specific sprint training method as it directly reflects the “traditional” sport-specific sprint pattern, which typically occurs under “unloaded conditions” (Nicholson et al., 2021; Zabaloy et al., 2023). Likewise, resistance training (RT) is also considered a complementary (i.e., tertiary) and essential sprint training method; although it does not replicate the traditional sprint pattern, it provides a specific neuromuscular stimulus and evokes key adaptations for sprint performance (Zabaloy et al., 2023).

Despite numerous studies comparing RST using a wide range of loads (e.g., from 10% to 135% body mass [BM]) and UST, there is still no clarity on the most appropriate loading range to increase sprint performance while avoiding negative changes in maximum velocity (V_max_) (Matusinski et al., 2021; Zabaloy et al., 2023). In this regard, Cahill et al. (2020) reported that heavy loading conditions (i.e., 135% BM) were particularly effective at improving acceleration over 5 and 10 m; nonetheless, these loads also resulted in a meaningful decrease (ES: 0.44) in V_max_. Similarly, other authors (Lahti et al., 2020; Morin et al., 2017) reported that heavy loads (i.e., ≥ 80% BM) resulted in meaningful increases in direct or indirect measures of sprint performance (i.e., split times, maximal theoretical force [F0] and velocity [V0]). Despite the relevance of these three studies (Cahill et al., 2020; Lahti et al., 2020; Morin et al., 2017), critical limitations were identified after careful analysis of the methods and design implemented. Briefly, 1) none of the three studies carefully considered the effects of RT programs on the outcomes of these respective interventions; 2) two of the studies (Lahti et al., 2020; Morin et al., 2017) implemented a combined training approach (RST + UST), with unresisted sprints accounting for more than 34% of the total training volume; 3) athletes in the unresisted conditions did not train at distances longer than 20 m, which may have hindered the potential for greater adaptations. In essence, it seems plausible that more robust and strictly controlled training designs may better clarify the importance of “heavy” loading conditions for sprint performance.

More recently, a study by Stavridis et al. (2023) reported that a 50% velocity decrement loading scheme (range: 58% to 72% BM) was an effective stimulus for inducing positive adaptations (e.g., F0, maximal horizontal power, velocity from 0–5 m to 25–30 m; range ∆%: 8.7 to 1.2) in sprint acceleration performance compared to UST and RST (10% BM) programs. However, the absence of detailed information on the athletes’ resistance training programs, the heterogeneity of the participant cohort (i.e., male and female athletes) and the distances used for training in UST and “light” loading condition (i.e., 20 m) may account for the lack of positive changes. Conversely, another recent study (Panasci et al., 2023) conducted with amateur male rugby players showed that a sled load of 12.6% BM induced positive effects on both acceleration and sprint performance. However, similarly to previous research (Cahill et al., 2020; Lahti et al., 2020; Morin et al., 2017), a combined approach (i.e., UST and RST) was employed and RT interventions were included in all groups. For this reason, the effectiveness of RST compared to UST on both acceleration and V_max_ performance is still widely debated, especially regarding the selection and prescription of the most appropriate and effective range of sled loads (e.g., light vs. heavy) to acutely or chronically enhance overall sprint performance (Matusinski et al., 2021, 2022; Panasci et al., 2023).

Although a recent meta-analysis (Murphy et al., 2023) revealed that several studies have analyzed the effects of RT regimes on sprinting capabilities, there is still a lack of information regarding the effects of sprint training programs on certain strength and power outcomes. As previously mentioned, since RT is a complementary method to sprint training, it is challenging to determine whether significant changes in strength- and power-related measures (e.g., isometric and eccentric strength, vertical and horizontal jumping abilities) can occur in the absence of structured RT (i.e., when reduced training volumes, frequency, and the number of lower limb exercises are performed), and when only RST or UST programs are systematically executed. In addition, it remains unclear whether reducing the volume and frequency of RT may affect strength- and power-related capacities in youth rugby union players.

Therefore, this study aimed to: 1) compare the effects of two different 4-week sprint training protocols (UST vs. RST at 50% BM) with equated volume, performed three times per week, on both resisted and unresisted sprint performance; 2) examine the effects of these sprint training programs on multiple lower limb strength measures (i.e., one-repetition maximum [1RM] in the squat exercise, and isometric squat test [ISqT] maximum force, eccentric hamstring strength in the Nordic hamstring exercise [NHE], and vertical and horizontal jump distances). Based on the principle of specificity, it was hypothesized that UST would effectively improve unresisted sprint velocity, while RST would have positive effects on maintaining or improving sprint velocity under resisted conditions. Finally, both UST and RST were expected to provide adequate stimuli to maintain strength- and power- related capacities across the different resistance exercises used in the current study.

## Methods

### Participants

Thirty-five under-19 male academy rugby union players (RST: n = 13; age: 17.3 ± 0.9 years; body mass: 79.2 ± 11.2 kg; body height: 1.77 ± 0.4 m; UST: n = 11; age: 17.4 ± 1.0; body mass: 82.8 ± 11.9; body height: 1.77 ± 0.6 m) were initially recruited to participate in this study. Participants had ≈10 years of training and playing experience and were accustomed to regularly perform sprint velocity training (i.e., UST and RST [loads range: 10–50% BM]) and RT blocks prior to the technical-tactical sessions. However, prior to the period of the study, they had not recently participated in any type of the RST program. Rugby players competed in the highest standard Argentinean junior league (i.e., Buenos Aires Rugby Union Top 12 competition) and participated in ≈6 weekly training sessions at their local rugby club, with one match per week. For the sake of interest, Argentina is currently the fifth highest-ranked team in the Men’s “World Rugby” rankings. To be eligible for the study, athletes had to be free of any musculoskeletal injuries for at least 6 months, as confirmed by the medical staff. In addition, a 100% completion rate was required for inclusion in the data analysis. Therefore, from the initial 35 participants, 11 were excluded from the study: three due to injuries sustained during competitions (e.g., concussions), two due to illness during the final training week (e.g., flu and fever), and the remaining six players for missing more than one training session due to personal reasons. The research was approved by the Ethics Committee of the Federal University of São Paulo (protocol code: 6.621.221; approval date: 23 January 2024), and all participants and their legal guardians provided informed consent prior to participation.

### Design and Procedures

This study was a randomized controlled trial analyzing two different sprint training methods: RST and UST. The experimental protocol was conducted at the beginning of the in-season period. Due to their regular training and measurement routines, all athletes were already familiarized with the tests and exercises used during the intervention period. A more detailed description of the 3-day testing schedule, including the sequence (i.e., order) of the measurements is provided in Table 1. In both pre- and post-intervention tests, each participant completed two maximal unresisted and one resisted (50% BM) 30-m sprint. Sprint performance variables (i.e., 10 m, 30 m and V_max_) were tested under both loading conditions, before and after the training intervention. All sprint training sessions were conducted at the same time of the day (i.e., 4.30 to 6.30 p.m.) at the club’s outdoor training facilities on a natural astro-turf rugby pitch. For pre- and post-training testing of lower limb strength, participants were assessed for the squat 1RM and ISqT, hamstring eccentric strength using the NHE, and jumping ability (countermovement [CMJ] and standing long jump [SLJ]). Once testing sessions were completed, participants were matched based on their best 30-m unresisted sprint times and randomly divided into two training groups (i.e., UST and RST at 50% BM). All testing sessions were conducted by the same experienced evaluators. Prior to each session, athletes were required to abstain from caffeine and alcohol and were not permitted to engage in any form of intense training 24 hours before testing. Similarly, before each pre- and post-training session, participants completed a general standardized warm-up consisting of 5 min of submaximal intensity cycling, followed by joint mobility exercises and a combination of skipping, high-knees, butt-kicking drills, forward lounges, free-weight deep squats, and plank variations. Afterwards, participants performed a specific warm-up (e.g., submaximal attempts of jumping and sprinting drills) prior to each testing session.

**Table 1:** Testing schedule for pre- and post-assessments of male academy rugby players.

*Day 1: Sprint Testing*	*Day 2: Strength Testing*	*Day 3: Jump and Hamstring Strength Testing*
4.30 p.m. (Group 1) 20-min Warm-up Unresisted sprints: 2 sets x 30 m (3-min recovery) Resisted sprint: 1 set x 30-m sled sprinting (50% BM) 5.30 p.m. (Group 2) 20-min Warm-up Unresisted sprints: 2 sets x 30 m (3-min recovery) Resisted sprint: 1 set x 30-m sled sprinting (50% BM)	5 p.m. (Group 1) 10-min Warm-up 3 repetitions of the Isometric Squat (5-min recovery) 1-RM Squat 6 p.m. (Group 2) 10-min Warm-up 3 repetitions of the Isometric Squat (5-min recovery) 1-RM Squat 7 p.m. (Group 3) 10-min Warm-up 3 repetitions of the Isometric Squat (5-min recovery) 1-RM Squat	5 p.m. (Group 1) 10-min Warm-up 2 repetitions of the SLJ 2 repetitions of the CMJ (3-min recovery) Hamstring Eccentric Strength (NHE) 6 p.m. (Group 2) 10-min Warm-up 2 repetitions of the SLJ 2 repetitions of the CMJ (3-min recovery) Hamstring Eccentric Strength (NHE) 7 p.m. (Group 3) 10-min Warm-up 2 repetitions of the SLJ 2 repetitions of the CMJ (3-min recovery) Hamstring Eccentric Strength (NHE)

Note: To minimize long recovery periods and reduce testing times, players were divided into groups. On day one, each group comprised approximately 15 players, while on days two and three, each group consisted of 10 players. *Abbreviations: BM: body mass; 1-RM: one-repetition maximum; CMJ: countermovement jump; SLJ: standing long jump; NHE: Nordic hamstring exercise*

With regard to the protocol, players performed three sessions per week over a 4-week training period, consisting of a sprint velocity training block with either resisted sled pulling (RST with 50% BM) or UST (Table 2). Subsequently, players completed a RT block focused primarily on core and upper limb resistance exercises, and only one complementary lower limb exercise that did not involve large hip or knee flexion and extension (e.g., lunges, box step up, squats, deadlifts, etc.). These exercises were programmed to ensure that RT did not act as a confounding factor for velocity and strength outcomes. A more detailed description of the training programs is presented in Tables 3–5. Additionally, the rate of perceived exertion (RPE) was recorded after the rugby-specific training block to monitor the subjective efforts of both groups. The RPE was used to quantify and control the intensity of each training session, with each participant rating the intensity of the field session using a 0–10 scale in an Excel spreadsheet on Google Drive (Google Drive, Google, CA, USA), approximately 5–10 min after the training session (Sams et al., 2020).

**Table 2:** Sprint training programs implemented during the 4-week intervention period for the unresisted and resisted sprint training groups.

*Training Group*	*Week 1–Week 3*		
	Day 1	Day 2	Day 3
Unresisted Sprint Training (Total Volume: 685 m)	1 set x 20 m (2-min recovery) 2 sets x 30 m (2-min recovery)	3 sets x 20 m (2-min recovery) 2 sets x 30 m (3-min recovery)	2 sets x 40 m (4-min recovery) 2 sets x 10 m (1-min recovery)
		*Week 2–Week 4*	
	2 sets x 20 m (2-min recovery) 2 sets x 40 m (4-min recovery)	1 set x 15 m (1-min recovery) 1 set x 20 m (2-min recovery) 3 sets x 30 m (3-min recovery)	1 set x 10 m (1-min recovery) 2 sets x 20 m (2-min recovery) 3 sets x 30 m (3-min recovery)
		*Week 1–Week 3*	
Resisted Sprint Training (Total Volume: 685 m)	1 set x 20 m (2-min 30-s recovery) 4 sets x 15 m (2-min recovery)	3 sets x 20 m (2-min 30-s recovery) 4 sets x 15 m (2-min recovery)	1 set x 20 m (2-min 30-s recovery) 4 sets x 15 m (2-min recovery) 2 sets x 10 m (1-min 30-s recovery)
		*Week 2–Week 4*	
	3 sets x 20 m (2-min 30-s recovery) 6 sets x 10 m (1-min 30-s recovery)	3 sets x 20 m (2-min 30-s recovery) 3 sets x 15 m (2-min recovery) 2 sets x 10 m (1-min 30-s recovery)	2 sets x 20 m (2-min 30-s recovery) 4 sets x 15 m (2-min recovery) 4 sets x 10 m (1-min 30-s recovery)

**Table 3:** Typical weekly training schedule for unresisted and resisted sprint training groups during the 4-week intervention period for male academy rugby players.

Sunday	Monday	Tuesday	Wednesday	Thursday	Friday	Saturday
Competition	Optional RT Block (30′): *Core training 10′* Upper body (20’): *Traditional exercises for pushing and pulling (BP, BO Row, Pull-ups, SP)* Recovery Block (30’): *Lower-limb stretching and foam roller*	Sprint Training Block (30’): *UST or RST (50% BM)* RT (60′): Lower body: *Non-Traditional exercises: Calf raises, Copenhagen Plank, Lateral Lunge* Upper body: *BP, Pull-ups, SP, BO row, LM Push, LM Row* TEC/TAC (90′)	Rest	Sprint Training Block (30’): *UST or RST* *(50% BM)* RT (60′): Lower body: *Non-Traditional exercises: Calf raises, Copenhagen Plank, Lateral Lunge* Upper body: *BP, Pull-ups, SP, BO row, LM Push, LM Row* TEC/TAC (90′)	Sprint Training Block (30’): *UST or RST (50% BM)* RT (40′): Upper body: *BP, Pull-ups, SP, BO row* TEC/TAC (60′)	Rest

Abbreviations: UST: unresisted sprint training; RST: resisted sprint training; RT: resistance training; BM: body mass; BP: bench press; BO Row: bent-over row; SH: shoulder press; LM: landmine; TEC/TAC: technical and tactical training session

**Table 4:** Resistance training program prescribed during the 4-week intervention period for male academy rugby players.

*WEEK 1 - WEEK 3*											
*DAY I*		*VOL: 80 m*		*DAY II*		*VOL: 120 m*		*DAY III*		*VOL: 100 m*	
*BLOCK 1*	SPRINT SPEED			*BLOCK 1*	SPRINT SPEED			*BLOCK 1*	SPRINT SPEED		
RESISTED OR UNRESISTED SPRINT BLOCK											
*BLOCK 2*	*STRENGTH/POWER*			*BLOCK 2*	*STRENGTH/POWER*			*BLOCK 2*	*STRENGTH/POWER*		
Lateral Lunge DB			3s x 6r (12)	Copenhagen Plank Iso Hold 6’’			3s x 6r (12)	SL weighted Planck			3s x 6r (12)
Bench Press BB			R: 45’’	Pull-Ups			R: 45’’	TRX Row			R: 45’’
			R: 2’				R: 2’	Military Press BB (SS)			R: 2’
Shoulder Press LM			3s x 6r (12)	Step-Up 20-cm Plate (50%BM)			3s x 6r (12)	SA Cable Lift (SS)			
LM BO Row			R: 45’’	Inclined Bench Press DB			R: 45’’				
Isometric Neck w/bands			R: 2’	Calf Raises Iso Hold DB			R: 2’	Cable Pullover			3s x 8r (12)
								Biceps Curl DB			R: 45’’
								Elbow Extension (Cable)			R: 2’
*BLOCK 3*	*COMPLEMENTARY UB*			*BLOCK 3*	*COMPLEMENTARY UB*			*BLOCK 3*	*RECOVERY*		
Lateral Raises DB			3s x 10r (15)	Declined Push-Ups			3s x 10r (15)	FOAM ROLLER/STRETCHING			
Arnold Shoulder Press DB			R: 45’’	SA BO Row DB			R: 45’’				
Dips			R: 2’	Posterior Raises Bands			R: 2’				
Biceps Hammer Curls				Biceps Curl BB							

Note: 1)Objectives: Maintenance of strength and power levels, development of acceleration and maximum velocity; 2) Loads: ~50% up to ~80% 1RM; 3) Level of effort: Medium; 4) Duration/Frequency: 4 weeks/3 times per week; SL/SA: repetitions are performed on both sides. Abbreviations: s: sets; r: repetitions; R: recovery; UB: upper body; DB: dumbbells; BB: barbell; LM: landmine; BO: bent-over; SA: single arm; SL: single leg; SS: split stance

**Table 5:** Resistance training program prescribed during the 4-week intervention period for male academy rugby players.

*WEEK 2 - WEEK 4*												
*DAY I*		*VOL: 120 m*		*DAY II*		*VOL: 125 m*			*DAY III*		*VOL: 140 m*	
*BLOCK 1*	*SPRINT SPEED*			*BLOCK 1*	*SPRINT SPEED*				*BLOCK 1*	*SPRINT SPEED*		
RESISTED OR UNRESISTED SPRINT BLOCK												
*BLOCK 2*	*STRENGTH/POWER*			*BLOCK 2*	*STRENGTH/POWER*				*BLOCK 2*	*STRENGTH/POWER*		
Bench Press BB			3s x 6r (12)	Lateral Lunge DB				3s x 6r (12)	Lateral Planck + Abduction			3s x 6r (12)
SL Calf Raises DB			R: 45’’	Pull-Ups				R: 45’’	BO Row DB			R: 45’’
			R: 2’					R: 2’	Military Press BB (Split Stance)			R: 2’
Shoulder Press LM			3s x 6r (12)	¼ Squat DB				3s x 6r (12)	Sit Up BB			
Pull-Ups (close grip)			R: 45’’	Inclined Bench Press BB				R: 45’’				
Iso Neck w/bands			R: 2’	TRX Row				R: 2’	Lateral Raises DB			3s x 8r (12)
									Cable Biceps Curl (Rope)			R: 45’’
									Push-Ups Narrow Grip			R: 2’
*BLOCK 3*	*COMPLEMENTARY UB*			*BLOCK 3*	*COMPLEMENTARY UB*				*BLOCK 3*	*RECOVERY*		
Frontal Raises DB			3s x 10r (15)	Shoulder Press DB (Altern)			3s x 12r (15)		FOAM ROLLER/STRETCHING			
Seated Shoulder Press DB			R: 45’’	SA Cable Row (HK)			R: 45’’					
Dips			R: 2’	SA Frontal Raises Bands			R: 2’					
Biceps Hammer Curls				Biceps Curl BB								

Note: 1) Objectives: Maintenance of strength and power levels, development of acceleration and maximum velocity; 2) Loads: ~50% up to ~80% RM; 3) Level of effort: Medium; 4) Duration/Frequency: 4 weeks/3 times per week; SL/SA: repetitions are performed on both sides. Abbreviations: s: sets; r: repetitions; R: recovery; UB: upper body; DB: dumbbells; BB: barbell; LM: landmine; BO: bent-over; SA: single arm; SL: single leg; HK: half kneeling

#### Anthropometric Measurements and Body Composition

Body mass and height were measured using an electronic scale (HD-366, Tanita Corporation, Japan), and a height rod and a vertex (Rosscraft Innovations, Vancouver, Canada), respectively, following the standard protocols recommended by the ISAK (Norton, 2018).

#### Unresisted and Resisted Sprint Tests

Before testing, participants performed a warm-up protocol consisting of 5 min of low- intensity running, joint mobility exercises, multidirectional displacements, and submaximal sprints ranging from 10 to 30 m, lasting a total of 20 min (Zabaloy et al., 2022b). Following the warm-up, players performed two 30-m sprints with a 3-min recovery period between trials. Sprint times were recorded using single-beam timing gates set at hip height (≈ 0.90 m) and positioned at 0, the 10^th^, the 20^th^, the 25^th^ and the 30^th^ m (Chronojump, Boscosystem, Barcelona, Spain). Subsequently, athletes performed one 30-m resisted sprint, pulling a sled (Fenix, Argentina; sled mass: 18 kg) attached to their waists, with a load equivalent to 50% of their BM (Velocity decrement [%]: pre-test, 45.3 ± 4.9; post-test: 40.8 ± 4.5). For both conditions, participants started in a two-point staggered stance just behind a line 0.5 m from the first gate and sprinted with maximum effort until passing a 35-m cone to avoid early deceleration. Accumulated sprint times at the 10^th^ m (T10), the 20^th^ m (T20), the 25^th^ m (T25) and the 30^th^ m (T30) were recorded, with the best T30 used for further analysis. Maximum velocity (V_max_) under resisted and unresisted conditions was calculated using a method described elsewhere (Zabaloy et al., 2024). Wind speed was monitored during pre- and post-testing sessions using an anemometer (Bentech Science and Technology, GM816, China), ensuring that wind speed remained < than 1.5 m•s^−1^ for all trials. The relative and absolute reliability of all sprint-derived variables at both pre- and post-tests was assessed, with the Intraclass Correlation Coefficient (ICC) ranging from 0.711 to 0.977 and the Coefficient of Variation (CV) ranging from 1.24% to 3.30%.

#### Isometric Squat Test

The test commenced following a specific warm-up protocol as previously described (Sánchez-Medina et al., 2017). Before the ISqT, athletes performed 5 min of mobility exercises, two sets of 10 unloaded squats, 10 walking lunges, 10 gluteal bridges, and 10 s of plank variations. Afterwards, participants were positioned correctly for the test, on a standard squat rack adopting a knee angle of 90º (±5), as previously reported (Loturco et al., 2024), measured using a mobile app digital goniometer (Protractor, Examobile, Poland). Once positioned, athletes completed the ISqT specific warm-up, which involved pushing against the bar for 3 s at 70–80% effort, with 1-min recovery between efforts. The same bar height was used in the post-training test for each athlete by controlling knee angles. During the measurements, after the start command, participants applied force as rapidly as possible against a fixed bar for 5 s. For each trial, participants were instructed to “push as hard and as fast as you can” to ensure maximal force was applied (Collings et al., 2024). Following previous guidelines (Collings et al., 2024; Kraska et al., 2009), athletes were required to get ready, followed by a countdown of “3, 2, 1, push!”. The “get ready” instruction required the participant to remove the slack from the “system” with minimal pretension, visually observed by an experienced evaluator (i.e., stable force trace at 100 N above the athlete’s bodyweight). Participants completed two maximal trials, with a third trial performed if there was a difference of ≈ 250 N between efforts (Collings et al., 2024; Kraska et al., 2009). Peak force (PF) was determined using portable, uniaxial dual force plates (Force Decks, FDLite V.2, VALD, Brisbane, Australia) (Collings et al., 2024) sampling at 1,000 Hz. The force plate was fixed to the floor using a standardized base. Strong verbal encouragement was provided throughout all attempts. The following ICCs and CVs were obtained: pre-test PF ICC: 0.948 and CV: 3.03% and post-test PF ICC: 0.975 and CV: 3.75%.

#### Squat 1RM

The 1RM-SQ was determined using a linear position transducer (Chronojump, Boscosystem, Barcelona, Spain) to measure movement velocity. The squat exercise was performed using a multi-rack and a 20-kg Olympic barbell (Fenix, Argentina). Participants started the test from an upright position, descending at a controlled velocity until their thighs surpassed the horizontal plane, with the barbell resting freely on the upper back, and were then required to perform concentric actions at maximal intended velocity (Sánchez-Medina et al., 2017; Zabaloy et al., 2022b). The initial load for the squat exercise was set at 40 kg and gradually increased by 5 to 10 kg. Approximately two to three repetitions were performed with each load, with 3-min rest intervals between sets. The test concluded when participants reached a mean propulsive velocity close to 0.5–0.6 m•s^−1^ (i.e., 80–85% 1RM), and subsequently the absolute 1RM was estimated using a previously published formula (Sánchez-Medina et al., 2017).

#### Vertical and Horizontal Jump Tests

Before testing, participants performed a specific warm-up of approximately 10 min, consisting of unloaded lunges and squats, and three submaximal sets of two repetitions of the CMJ with hands on the hips. In the CMJ, players were instructed to perform a downward movement followed by complete extension of the lower limbs and the amplitude of the countermovement was freely determined to avoid changes in jumping coordination (Loturco et al., 2022). Three attempts of CMJs were performed with 15-s intervals, and a 2-min rest was allowed between trials. The jumps were assessed using portable, uniaxial, dual force plates (Force Decks, FDLite V.2, VALD, Brisbane, Australia) (Collings et al., 2024) and the best attempt (i.e., highest jump height) was used for further analysis. For the standing long jump (SLJ), participants performed two maximal horizontal jumps with hands on their hips, separated by 15 s. Athletes were verbally encouraged to jump as far as possible while landing in a controlled manner (i.e., bouncing or losing control upon landing was not permitted). Jump distance was measured with a measuring tape from the starting line to heel closest to the line, with the longest distance attained used for subsequent analysis. The following ICCs and CVs were obtained: pre-test CMJ ICC: 0.981 and CV: 2.41% and pre-test SLJ ICC: 0.978 and CV: 2.16%; post-test CMJ ICC: 0.966 and CV: 3.12% and post-test SLJ ICC: 0.948 and CV: 3.34%.

#### Nordic Hamstring Exercise Test

Participants performed the NHE on an instrumented Nordbord (Vald Performance, Australia) sampling at 400 Hz. This portable device has demonstrated moderate to high levels of test-retest reliability during bilateral testing and offers an alternative to current dynamometry-based techniques for assessing eccentric knee flexor strength (Opar et al., 2013). Briefly, the NHE is a bodyweight exercise that requires athletes to start in a kneeling position and gradually lower their upper body and thighs toward the ground by extending at the knee, while eccentrically contracting the knee flexors to slow the descent (Opar et al., 2012). In the starting position, participants kneeled on the padded part of the NordBord, with the upper body in an upright position aligned with thighs, and their ankles secured against the padded hooks (Ruan et al., 2021). Participants were required to gradually lean forward by contracting the hamstrings, while keeping their trunk and hips in a neutral position throughout. Instructions to the players followed previous recommendations (Buchheit et al., 2016). Specifically, they were required to lean forward as slowly as possible while maximally resisting the movement with both limbs, keeping their trunk and hips in a neutral position and their hands across the chest. Once participants reached the end of the eccentric phase, they were asked to slowly return to the initial kneeling position to avoid additional force traces being considered. Participants completed one set of three maximal repetitions, and the mean maximum force (MMF) was used for analysis.

### Statistical Analysis

Intrasession relative and absolute reliability was examined using the ICC two-way random effects model (95% confidence interval [CI]) and the CV was calculated following previous guidelines (Koo and Li, 2016). Anderson-Darling tests verified normality of all pre-test measures for both groups, as well as the delta values (post minus pre) for all measures within groups (*p* > 0.05). Descriptive statistics (mean ± SD) were calculated for all pre- and post-test measures. Between-group pre-test differences were assessed using independent samples *t*-tests after confirming homogeneity of variances with the Levene’s test for equality of variances. Within-group pairwise comparisons (pre vs. post) were performed using paired samples *t*-tests. Hedges’ g effect size (ES) with the 95% CI was used to quantify the strength of the observed pairwise differences. The Hedges’ g ES was chosen as it corrects for bias in small sample sizes; magnitudes were interpreted as follows: trivial (< 0.20), small (0.20–0.49), moderate (0.5–0.79), and large (> 0.80) (Cohen, 1988). One participant from the RST group did not complete the post-intervention testing for strength and jump measures; consequently, only their sprint data were retained for the analysis.

Differences between experimental groups in the pre- and post-differences were investigated by fitting a linear regression model with the post-test outcome variable as the dependent variable, the experimental group as the main independent variable, and the pre-test measure as a continuous covariate. After verifying model assumptions, the effect of the group (i.e., the mean difference between groups in the respective pre-post differences, adjusted for pre-test values) was estimated alongside the associated 95% CI. Estimated marginal means (± standard errors) were subsequently extracted for descriptive purposes. The Benjamini-Hochberg procedure was used to control the false discovery rate at 5% due to the number of hypotheses tested (Benjamini and Hochberg, 1995). This procedure was applied to the alpha level in both the primary analyses (i.e., within- and between-group analyses of the primary outcome variables: 10- and 30-m sprint times) and the secondary analyses separately (Feise, 2002). All statistical analyses were performed using R 4.4.1 software (R Core Team 2024) in RStudio (2024.4.2.764).

## Results

Comparison of the RPE between groups showed no significant differences across the intervention period (RST: 6.0 ± 0.7 and UST: 6.3 ± 0.7; *p* = 0.470, ES: 0.29). Additionally, none of the primary or secondary outcome measures were significantly different at pre-test (*p* > 0.05). Within-group comparisons from the pre- to post-training time points for the primary and secondary outcome measures are reported in Table 6.

**Table 6:** Pre- and post-training within-group comparison of unresisted and resisted sprints, as well as strength and jump performance measures, in male academy rugby players.

Variable	Resisted Sprint Training				Unresisted Sprint Training			
	Pre	Post	Hedges’ g [95% CI]	*p-*value	Pre	Post	Hedges’ g [95% CI]	*p-*value
*Primary Outcomes*								
Unresisted 0–10 m (s)	1.91 ± 0.08	1.90 ± 0.13	0.18 [−0.33; 0.70]	0.491	1.91 ± 0.07	1.87 ± 0.07	0.53 [−0.08; 1.11]	0.088
Unresisted 0–30 m (s)	4.46 ± 0.17	4.41 ± 0.21	0.52 [−0.04; 1.06]	0.067	4.43 ± 0.15	4.34 ± 0.12	0.93 [0.24; 1.59]	0.008*
*Secondary Outcomes*								
Body Mass (kg)	79.2 ± 11.2	79.9 ± 10.7	−0.43 [−0.96; 0.11]	0.122	82.8 ± 11.9	83.2 ± 12.3	−0.40 [−0.97; 0.18]	0.179
V_max_ (m•s^−1^)	8.41 ± 0.45	8.44 ± 0.52	−0.12 [−0.63; 0.40]	0.658	8.46 ± 0.52	8.44 ± 0.36	0.09 [−0.46; 0.63]	0.765
Resisted 0–10 m (s)	2.99 ± 0.19	2.79 ± 0.16	1.35 [0.60; 2.08]	<0.001*	2.95 ± 0.12	2.72 ± 0.15	1.24 [0.46; 1.99]	0.001*
Resisted 0–30 m (s)	7.39 ± 0.46	6.93 ± 0.54	1.33 [0.58; 2.05]	<0.001*	7.19 ± 0.34	6.70 ± 0.35	1.82 [0.85; 2.77]	<0.001*
Resisted V_max_ (m•s^−1^)	4.66 ± 0.33	5.09 ± 0.55	−0.90 [−1.50; −0.26]	0.005*	4.86 ± 0.35	5.18 ± 0.32	−1.28 [−2.05; −0.49]	0.001*
Isometric Peak Force (N)	2225 ± 405	2468 ± 300	−0.90 [−1.53; −0.24]	0.006*	2296 ± 365	2641 ± 570	−0.68 [−1.29; −0.05]	0.034
Squat 1RM (kg)	121.8 ± 20.0	122.3 ± 22.2	−0.06 [−0.58; 0.47]	0.832	132.9 ± 23.8	135.4 ± 28.6	0.27 [−0.29; 0.82]	0.350
Standing Long Jump (m)	1.93 ± 0.16	1.99 ± 0.17	−0.83 [−1.44; −0.19]	0.011*	1.99 ± 0.21	2.04 ± 0.20	−0.65 [−1.25; −0.03]	0.041
CMJ Height (m)	0.385 ± 0.036	0.391 ± 0.038	−0.28 [−0.81; 0.27]	0.323	0.388 ± 0.053	0.393 ± 0.045	−0.21 [−0.76; 0.35]	0.468
Left Hamstring Maximum Force (N)	328 ± 81	387 ± 63	−0.90 [−1.53; −0.24]	0.007*	404 ± 100	421 ± 79	−0.35 [−0.91; 0.22]	0.234
Right Hamstring Maximum Force (N)	320 ± 84	386 ± 63	−1.10 [−1.79; −0.39]	0.002*	391 ± 76	415 ± 67	−0.53 [−1.11; 0.07]	0.086

Note: Data are presented as means ± standard deviations (SD); *< α. After adjustment, two p-values were no longer below α: URS Iso peak force (p = 0.034) and URS standing long jump (p = 0.041). All other highlighted values remain significant. Abbreviations: V_max_: maximum velocity; 1RM: one-repetition maximum; CMJ: countermovement jump

**Figure 1:**
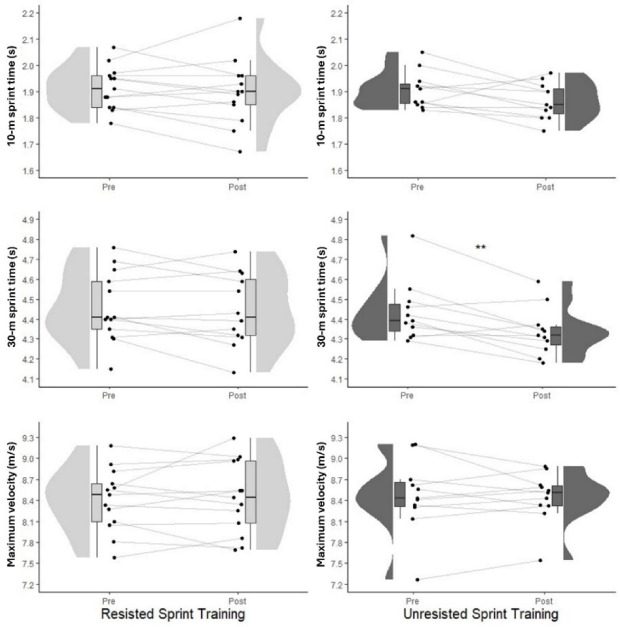
Pre- and post-training changes in 10-m sprint time (top), 30-m sprint time (middle), and maximum velocity (bottom) for the Resisted Sprint Training group (RST; left) and the Unresisted Sprint Training group (UST; right).

Regarding BM, no changes were detected for any of the groups, neither within nor between groups at any time points assessed (*p* > 0.122; ES: trivial to small). Concerning sprint performance, a large significant reduction in T30 (*p* = 0.008) was found following the UST intervention. Conversely, T10 and V_max_ remained unchanged (*p* > 0.08; ES: trivial to moderate) in this group and no differences were observed in unresisted sprint times (T10 and T30) and V_max_ (*p* > 0.067; ES: trivial to moderate) following RST. Pre- to post-training changes in unresisted and resisted sprint times are illustrated in Figure 1.

For the secondary outcome measures, both groups showed significant improvements in resisted T10, T30 and V_max_ (*p* < 0.005; ES: large). Regarding strength, the RST group presented significant increases in ISqT PF, SLJ, and NHE maximum force in both the right and left legs (*p* < 0.011; ES: large). No significant changes were observed in the remaining variables (i.e., squat 1RM and CMJ) in either group.

Between-group comparisons across all measures are presented in Table 7. Overall, no significant differences were detected between RST and UST in any of the primary or secondary measures following the 4-week intervention period.

**Table 7:** Post-training between-group comparison of unresisted and resisted sprint times, as well as strength and jump performance measures in youth rugby players.

Variable	Resisted Sprint Training	Unresisted Sprint Training	Between Group Difference [95% CI]	*p*-value
*Primary Outcomes*				
Unresisted 0–10 m (s)	1.89 ± 0.02	1.87 ± 0.02	0.03 [−0.04; 0.09]	0.421
Unresisted 0−30 m (s)	4.40 ± 0.03	4.35 ± 0.03	0.05 [−0.03; 0.13]	0.190
*Secondary Outcomes*				
Body Mass (kg)	81.5 ± 0.4	81.3 ± 0.4	0.3 [−0.8; 1.4]	0.626
V_max_ (m•s^−1^)	8.46 ± 0.07	8.41 ± 0.08	0.05 [−0.18; 0.27]	0.662
Resisted 0−10 m (s)	2.78 ± 0.03	2.73 ± 0.04	0.06 [−0.06; 0.17]	0.334
Resisted 0−30 m (s)	6.85 ± 0.08	6.79 ± 0.09	0.06 [−0.20; 0.32]	0.636
Resisted V_max_ (m•s^−1^)	5.16 ± 0.10	5.09 ± 0.11	0.07 [−0.26; 0.40]	0.671
Isometric Peak Force (N)	2493 ± 105	2614 ± 109	−121 [−437; 195]	0.432
Squat 1RM (kg)	128.1 ± 2.5	128.9 ± 2.6	−0.8 [−8.5; 6.9]	0.829
Standing Long Jump (m)	2.02 ± 0.02	2.01 ± 0.02	0.02 [−0.04; 0.08]	0.572
CMJ Height (m)	0.393 ± 0.06	0.391 ± 0.06	0.01 [−0.16; 0.18]	0.884
Left Hamstring Maximum Force (N)	410 ± 13	396 ± 14	14 [−27; 55]	0.482
Right Hamstring Maximum Force (N)	408 ± 13	391 ± 13	17 [−23; 57]	0.380

Note: Data are presented as adjusted means ± standard errors (adjusted for baseline values). Abbreviations: V_max_: maximum velocity; 1RM: 1-repetition maximum; CMJ: countermovement jump

## Discussion

The present study aimed to analyze: 1) the effects of two different sprint training methods (UST vs. RST at 50%BM) with equated volume over a 4-week training period on resisted and unresisted sprint performance; 2) the effects of these sprint protocols on various lower limb strength measures. It is important to note that none of the strength exercises tested were included in the training sessions during the intervention period, and the lower limb RT volume and intensity were kept to a minimum throughout the 4 weeks (i.e., 2–3 sets of 4–6 reps of 2–3 exercises performed at 40–60% 1RM, twice per week). The main findings indicate that only the UST condition provided a sufficient stimulus to reduce unresisted T30 sprint times. The significant reductions observed (p = 0.008; ES: 0.93) in the UST group contrast with the non-significant (p = 0.067; ES: 0.52) reductions in the RST group. In addition, unresisted T10 and Vmax remained unchanged across both training conditions. Notably, both methods (i.e., UST and RST) showed significant (p < 0.005; ES: 0.90 to 1.82) positive changes in resisted performance across all measures (i.e., T10, T30, and Vmax). Lastly, while both sprint methods were a sufficient training stimulus to maintain maximum strength and vertical jump capacities, only RST participants demonstrated superior ISqT (i.e., PF) and NHE eccentric strength (i.e., MMF) in both, left and right limb measures after the 4-week period. However, the between-group analysis (adjusted for baseline) showed no superiority of one sprint training method over another in improving sprint or strength performance.

Regarding the main outcome measures (i.e., T10, T30, and Vmax), the present findings highlight the importance of implementing UST to induce positive adaptations to sprint performance as seen by the significantly faster T30 only in the unloaded condition, without adverse modifications in T10 and Vmax. Conversely, RST at 50% BM was unable to improve unresisted sprint times (i.e., T10 and T30) and Vmax after the 4-week training period. These results partially align with a previous study (Panasci et al., 2023) on amateur rugby players, where the authors reported significant changes in T10 and T30 for both UST and RST at 12.6% BM. Similarly, in elite rugby players, another study (Sinclair et al., 2021) showed that URS and RST (loads: 20% velocity loss [≈ 25% BM]) were equally effective in improving unresisted sprint times, with no differences between the groups in terms of improvement. Despite the observed similarities with our study, it is worth noting that, in the current investigation: 1) RT was strictly controlled, which is a crucial aspect as RT is classified as a “tertiary sprint training method” (Zabaloy et al., 2023) that could be considered a confounding variable; 2) the RST intervention exclusively involved loaded sprint efforts, with no inclusion of unresisted sprinting (i.e., no combined training regime was employed), allowing for the isolation of the effects of one type of training (UST) from those of the other (RST) (Zabaloy et al., 2023). In fact, although previous research (Cahill et al., 2020; Lahti et al., 2020; Morin et al., 2017; Stavridis et al., 2023) across various athletic populations (i.e., college-level athletes, amateur, and professional soccer players) showed positive changes in sprint times from 5 to 20 m following heavy RST, these results were possibly influenced by several factors, such as the utilization of large volumes (> 35%) of UST or the lack of control over, or reduction of, lower limb RT program volumes. In this regard, Loturco et al. (2017) previously emphasized that mixed training approaches (i.e., RST combined with RT exercises or plyometrics) produced positive adaptations in different phases of sprinting in professional soccer players. Only Stavridis et al. (2023) demonstrated clear changes after heavy sled sprinting (i.e., 50% velocity loss) compared to light resisted (10% BM) or UST. However, for sprinters, a 20-m sprint stimulus may not be sufficient to induce positive adaptations when sprinting under light or unloaded conditions.

In terms of resisted sprint performance, somewhat surprisingly, both groups were equally effective to reduce T10 and T30 while increasing resisted Vmax. Based on these findings, one could argue for the usefulness of UST not only in improving unresisted sprint velocity, but also in enhancing sprint velocity under resisted conditions. The current findings show, for the first time, that RST (50% BM) is not superior in improving resisted sprint performance in youth rugby union players. In fact, the implementation of such loads seems unnecessary when the aim is to enhance sprint performance, as indicated by the results observed here. Moreover, Loturco et al. (2017) also reported that RST loads < 20% BM, when combined with the “optimum power load” (i.e., load that maximizes power output) during the jump squat, were effective for increasing resisted sprint velocities over various distances (from 5 to 20 m). In the present study, youth rugby players training under UST conditions demonstrated large positive changes across all resisted sprint measures. While the underlying mechanisms (i.e., physiological, physical, and biomechanical aspects) responsible for these adaptations were not analyzed, it seems plausible to suggest that a frequency of three sessions per week of sprint training, with volumes ranging from 80 m to 140 m per session and distances from 10 m to 40 m, induced the large and significant changes observed in the UST group.

Importantly, similar modifications were observed in RST with an identical frequency and volume, although the distances used for training under this loading scheme were shorter (i.e., 10 m to 20 m). In this sense, coaches and practitioners should consider the present findings, under both unresisted (primary outcomes) and resisted (secondary outcomes) sprint conditions, to better design sprint training programs. It is worth noting that a previous study (Pareja-Blanco et al., 2020) reported that moderate loads (i.e., 40% BM) improved performance in both unresisted and resisted sprinting across a wide range of loading conditions, from 20% to 80% BM. Nevertheless, the study by Pareja-Blanco et al. (2020) differs significantly from the present one, particularly in terms of participants (e.g., physically active women) and training frequency (i.e., once a week), making the results not directly comparable to those reported here.

The effects observed after the 4-week training period on strength performance showed that both sprint training conditions were effective in maintaining or even improving lower limb strength over time. To the best of our knowledge, this is the first study to analyze the effectiveness of a sprint training program on multiple strength and power measures in youth rugby players. Our results showed that UST was able to maintain various strength-related measures (i.e., 1RM SQ, ISqT PF, NHE MMF, CMJ and SLJ), despite none of these exercises being trained during the intervention period. Conversely, the RST group data revealed that towing a sled load of 50%BM was effective in increasing isometric force production (i.e., PF), eccentric maximum force in the NHE, and SLJ distance. Accordingly, Zabaloy et al. (2023) previously suggested that loads > 50% BM should be considered a “strength training stimulus” (i.e., a tertiary rather than a secondary method for developing sprint performance and enhancing sprint technique) due to the drastic modifications and disruptions that heavier loads produce on sprinting mechanics (Pareja-Blanco et al., 2022; Zabaloy et al., 2022a). Hence, when coaches aim to improve strength-power measures while reducing the frequency and volume of traditional RT programs (e.g., strength-power training programs) for the lower limbs, RST with 50% BM appears to be a viable option. More specifically, the improvements observed in ISqT PF are not surprising, given the associations reported between this explosive-isometric test and sprint performance in rugby and hurling athletes (Brady et al., 2024; Tillin et al., 2013). Additionally, since hamstring injuries are among the most common non-contact injuries in sports involving high-speed running (Opar et al., 2012), the improvements observed in eccentric MMF for both the left and right limbs during the NHE suggest that RST could serve as an effefctive training tool for reducing the risk of hamstring injuries. Lastly, it is important to note that UST also showed moderate increases in ISqT PF and the SLJ, although these values were not below α after adjustment and, therefore, cannot be considered significantly different from pre-test results. Nevertheless, it is relevant to note that, despite the within-group changes reported and discussed herein, no between-group differences were observed which, once again, does not allow us to advocate for the superiority of one approach over the other.

The current study has some limitations that must be acknowledged. Firstly, prescribing a load based on % BM does not account for individual variations in participants’ characteristics (i.e., strength and power capabilities, sprint velocity, anthropometric aspects, etc.) or surface friction (Stavridis et al., 2023). In contrast, it is important to highlight that this strategy is widely used in real-world training environments due to its practicality and ease of use. Secondly, the sample size and duration of the training period could have been larger. However, this is a common issue in studies involving top-level athletes in high-performance environments due to typical time-constraints and the limited number of available subjects (i.e., elite athletes) on a team.

## Conclusions

The findings of this study demonstrate that a 4-week training intervention, involving sprint training performed three times per week using two different methods (i.e., UST and RST), was effective in maintaining unresisted (i.e., T30 was reduced only in the UST group) and improving resisted sprint velocity in both groups of rugby union players. Additionally, RST (50% BM) was found to enhance explosive-isometric force production and eccentric hamstring strength, further supporting previous observations (Zabaloy et al., 2023) that identified heavy sled loads as a tertiary (i.e., complementary) sprint training method. In practical terms, practitioners could consider increasing the frequency and volume of speed training to reduce gym time and prioritize sprinting-focused training sessions. Since strength and power measures were maintained or even improved despite minimal or no lower limb RT, this approach may be especially advantageous for teams as they prepare for the final phases of the season. As a final point, coaches should be aware that the systematic implementation of RST programs, particularly under heavier loading conditions, might lead to increased levels of fatigue, potentially resulting in chronic decreases in both technical and physical performance over the course of the competitive season.
